# Effectiveness of physiotherapy exercise following hip arthroplasty for osteoarthritis: a systematic review of clinical trials

**DOI:** 10.1186/1471-2474-10-98

**Published:** 2009-08-04

**Authors:** Catherine J Minns Lowe, Karen L Barker, Michael E Dewey, Catherine M Sackley

**Affiliations:** 1 Department of Primary Care Clinical Sciences, University of Birmingham, Edgbaston, Birmingham, UK; 2 Physiotherapy Research Unit, Nuffield Orthopaedic Hospital NHS Trust, Windmill Road, Headington, Oxford, UK; 3 School of Community Health Sciences, University of Nottingham, University Park, Nottingham, UK

## Abstract

**Background:**

Physiotherapy has long been a routine component of patient rehabilitation following hip joint replacement. The purpose of this systematic review was to evaluate the effectiveness of physiotherapy exercise after discharge from hospital on function, walking, range of motion, quality of life and muscle strength, for osteoarthritic patients following elective primary total hip arthroplasty.

**Methods:**

*Design*: Systematic review, using the Cochrane Collaboration Handbook for Systematic Reviews of Interventions and the Quorom Statement.

*Database searches*: AMED, CINAHL, EMBASE, KingsFund, MEDLINE, Cochrane library (Cochrane reviews, Cochrane Central Register of Controlled Trials, DARE), PEDro, The Department of Health National Research Register. Handsearches: *Physiotherapy, Physical Therapy, Journal of Bone and Joint Surgery (Britain) Conference Proceedings*. No language restrictions were applied.

*Selection*: Trials comparing physiotherapy exercise versus usual/standard care, or comparing two types of relevant exercise physiotherapy, following discharge from hospital after elective primary total hip replacement for osteoarthritis were reviewed.

*Outcomes*: Functional activities of daily living, walking, quality of life, muscle strength and range of hip joint motion. Trial quality was extensively evaluated. Narrative synthesis plus meta-analytic summaries were performed to summarise the data.

**Results:**

8 trials were identified. Trial quality was mixed. Generally poor trial quality, quantity and diversity prevented explanatory meta-analyses. The results were synthesised and meta-analytic summaries were used where possible to provide a formal summary of results. Results indicate that physiotherapy exercise after discharge following total hip replacement has the potential to benefit patients.

**Conclusion:**

Insufficient evidence exists to establish the effectiveness of physiotherapy exercise following primary hip replacement for osteoarthritis. Further well designed trials are required to determine the value of post discharge exercise following this increasingly common surgical procedure.

## Background

Osteoarthritis is the commonest cause of disability in older people [1]. Prevalence figures for hip osteoarthritis range from 7–25% in people aged over fifty five [2] with over 70% of sufferers experience pain and limitations in performing activities of daily living, such as mobility outside the home [3]. Effective treatment exists for end-stage disease in the form of joint arthroplasty [4]. The number of primary total hip replacements procedures for osteoarthritis continues to rise steadily with 51,981 procedures reported for England and Wales in 2006 [5]. Traditionally, physiotherapy has been a routine component of patient rehabilitation following hip joint replacement. Due to the introduction of initiatives such as integrated care pathways, the length of hospital stay following joint replacement surgery has markedly and rapidly decreased [6], with the duration period for post operative in-patient physiotherapy being reduced. For patients without incapacitating systemic or life threatening disease the average length of stay in hospital between 2003–2006 was 7.4–8.9 days in England and Wales [6]. It is known that impairments and functional limitations remain a year after surgery [7] so the effectiveness of post discharge physiotherapy upon functional ability after hip replacement is a valid question. Current uncertainty regarding effectiveness makes it difficult for primary care commissioning organisations to determine whether to provide a post discharge physiotherapy service to patients, for primary health care practitioners advising patients regarding follow up after discharge and for patients making decisions about their own health care. Several general reviews of the literature surrounding rehabilitation following hip joint replacement exist [7-9] and one recent review, limited to Medline and Cochrane databases, for literature regarding physical training before and after hip and knee arthroplasty [10]. This narrative review was date limited (1996–2006), did not include physiotherapy/physical therapy as a search term, did not search other databases likely to contain allied health professional research records and did not identify the majority of trials included in this review. Since Dauty et al (1997) [10] considered physical training rather than physiotherapy practice there have not been, as yet, any systematic reviews exploring the effectiveness of post discharge physiotherapy following hip joint replacement surgery. We aimed to review, systematically, randomised controlled trials in order to answer the following question 'To what extent is post discharge physiotherapy exercise effective, in terms of improving function, quality of life, mobility, range of hip joint motion and muscle strength, for osteoarthritic patients following elective primary unilateral total hip arthroplasty?'

## Methods

### Ethical Approval

The Oxford Local Research Ethics Committee awarded approval for this study (AQREC No: A03.018).

### Searching

In March 2005 and April 2007 we identified clinical trials by simultaneously searching AMED (from 1985), CINAHL (from1982), EMBASE (from1974), KingsFund Database (from1979) and MEDLINE (from 1966). The Cochrane library, PEDro physiotherapy evidence database and The Department of Health National Research Register were also searched. In July 2005 and April 2007 we hand searched *Physiotherapy *(1985–March 2007 inclusive), *Physical Therapy *(1985–April 2007 inclusive) to double check for trials. The conference proceedings in the *Journal of Bone and Joint Surgery (Britain) *(1985–2006 inclusive) were also handsearched, as were the reference lists of included trials. The location of Physiotherapy trials is difficult therefore, although time consuming, multiple general searches were considered the optimum location method. This review is part of a series with both knee and hip search terms being included. Searches are summarised in Table 1. No language restrictions were applied. Professional translation of non English language articles was obtained using a translation service familiar with medical terminology.

**Table 1:** Search Strategy for Systematic Review.

Source	Searches and Search Terms	Mar–Jul 2005 Hits* (number of new relevant records)	2005–April 2007 Hits* (number of new relevant records)
KA24: AMED 1985 - CINAHL 1982- EMBASE 1974- Kingsfund 1979- MEDLINE 1966-	1. "hip" OR "knee" (whole document) AND "replacement" OR "arthroplast$" (whole document) AND "rehabilitation" AND "trial$" (whole document)	587 (25)	180 (0)
			
	2. "hip" OR "knee" (whole document) AND "replacement" OR "arthroplast$" (whole document) AND "rehabilitation" AND "trial$" (title)	118 (11)	1 (0)
			
	3. "hip" OR "knee" (whole document) AND "replacement" OR "arthroplast$" (whole document) AND "physiotherapy" AND "trial$" (title)	2 (0)	4 (0)
			
	4. "hip" OR "knee" (whole document) AND "replacement" OR "arthroplast$" (whole document) AND "physiotherapy" (title)	39 (0)	14 (0)
			
	5. "hip" OR "knee" (whole document) AND "replacement" OR "arthroplast$" (whole document) AND "physical therapy" (title)	43 (8)	15 (0)
			
	6. "hip" OR "knee" (whole document) AND "replacement" OR "arthroplast$" (whole document) AND "home programme" (title)	2 (0)	1 (0)
			
	7. "hip" OR "knee" (whole document) AND "replacement" OR "arthroplast$" (whole document) AND "home programme" (whole document)	22 (2)	27 (0)
			
	8. "hip" OR "knee" (whole document) AND "replacement" OR "arthroplast$" (whole document) AND "occupational therapy " (whole document)	35 (0)	3 (0)
			
	9. "hip" OR "knee" (whole document) AND "occupational therapist$" (title)	0 (0)	3 (0)

Cochrane library: Cochrane reviews CCRCT DARE	1. Browsed by topic musculoskeletal Search narrowed osteoarthritis Search narrowed rehabilitation	9	11
	2. General search term "joint replacement"	80	18

PEDro physiotherapy evidence database	1. "joint replacement AND rehabilitation"	1 (0)	17 (0)
	2. "joint replacement"	5 (0)	45 (0)

Dept of Health National Research Register	1. "joint replacement AND rehabilitation"	0	19 (1)
	2. "joint replacement AND physiotherapy"	7	9 (0)
	3. "joint replacement AND exercise"	2 (0)	6 (0)
	4. "joint replacement AND physical therapy"	3 (0)	5 (0)
	5. "joint arthroplasty AND physiotherapy"	0	0
	6. "joint arthroplasty AND rehabilitation"	2 (1)	2 (0)
	7. "joint replacement AND occupational therapy"	5 (0)	5 (0)

Physiotherapy	Key journal – Hand search of contents pages	Nil new	Nil new

Physical Therapy	Key journal – Hand search of contents pages	Nil new	Nil new

JBJS [Br]	Hand search of all conference proceedings	2 new	1

Reference lists	Hand searching of papers included in the review	1 new.	

Totals		965 (50)	386 (2)

* Numbers following use of removal of duplicates commands when available.

### Selection

We sought prospective comparative clinical trials of patients undergoing total hip replacement for osteoarthritis who received a physiotherapy exercise rehabilitation intervention following discharge from hospital post-operatively. We used broad definitions of "physiotherapy" and "exercise" to include any exercises or exercise programme advised or provided by physiotherapists/physical therapists during the rehabilitative period after discharge from hospital after surgery occurring in the out patient, community or home setting. This is not to say other forms of exercise are considered lesser in any way, only that our area of interest was physiotherapy practice. Trials were included if they compared a physiotherapy intervention versus usual or standard care or compared two different types of relevant physiotherapy intervention. We excluded trials in which the intervention consisted of an electrical adjunct to physiotherapy. Effectiveness outcomes included in trials were measures of functional activities of daily living, walking, self report measures of quality of life, muscle strength and range of hip joint motion. As most trials use functional measures, which include pain, rather than specific pain outcomes, it was not considered possible to include pain as a separate effectiveness outcome. Study eligibility was assessed and agreed by two reviewers (CML and CS).

### Validity assessment, data abstraction and quality assessment

We developed and piloted a data extraction form, using quality indicators from the CONSORT statement [11] and the CASP guidelines [12] (Table 2). Similar analysis of individual quality components is a previously used approach within physiotherapy reviews [13,14] and is advocated to avoid known problems associated with existing composite scores [15]. Items could be marked as yes, no, unclear or partial. Items were only marked as yes if they fully and explicitly met the detailed criteria laid out in the CONSORT standards [11]. Two non English language trials were identified and translated, one of which was written up in summary form rather than as a journal paper and could not be included in this assessment of quality for the review [16]. Two reviewers independently extracted the data (CML and KB). KB was masked to the key details of each English language paper and the extent to which masking was successful was assessed. The two non English trials were excluded from this masked monitoring process since the translations arrived in non journal format after this process was completed. The masking rates were 83.33% for authors, 33.33% for journals, and 100% for author affiliations, funding sources, and study location; all rates were considered successful bar journal of publication. The level of agreement between reviewers, using the component checklist, was 70.45%, (kappa 0.570, intraclass correlation coefficient (2,1) 0.699 (95% CI 0.606 to 0.770).

**Table 2:** Quality component checklist and quality evaluation of seven trials included in the review (Kaae *et al*.,1989 excluded*).

Does the study/author information adequately contain the following:	Jan *et al*., 2004	Johnsson *et al*., 1988	Nyberg & Kreuter, 2002	Patterson *et al*., 1995	Sashika *et al*., 1996	Suetta *et al*., 2004	Trudelle-Jackson & Smith 2004
Rationale for study	Y	Y	Y	Y	Y	Y	Y

Eligibility criteria	Y	P	Y	Y	Y	Y	Y

Recruitment method	N	N	P	P	Y	P	Y

Settings and location of study	P	P	Y	Y	P	P	P

Intervention	Y	P	Y	P	Y	P	Y

Objectives/hypotheses	P	P	N	P	P	Y	Y

Defined outcome measures	Y	P	N	N	P	Y	Y

Quality enhancers (e.g. multiple observations)	Y		N	P	N	P	Y

Sample size determination	Y	N	N	N	P	Y	N

Randomisation	Alternately assigned	Y	Alternately assigned	Assigned by location	Not randomised	Y	Y

Randomisation sequence generation	Y	N	Y	Y	I	Y	Y

Allocation concealment	N	N	Y	N	N	N	N

Randomisation implementation methods	N	N	N	N	N	N	N

Blinding – participant	N	N	N	N	N	N	Y

Blinding – of those administering the intervention	N	N	N	N	N	P	N

Blinding – outcome/assessments	Y	N	Y	Y	N	Not all blinded	Not blinded

Statistical methods	Y	P	Y	Y	N	Y	Y

Flow of participants through each stage	Y	U	Y	N	P	P	Y

Recruitment and follow up dates	N	N	Y	N	N	P	N

Baseline demographics	Y	N	P	Y	Y	Y	Y

Numbers analysed (and ITT)	Y	U	P	U	Y	P	Y

Summary of Results	P	P	P	Y	P	Y	Y

Estimated effect sizes	N	P	N	N	N	N	N

Precision	N	N	N	Y	N	Y	N

Results for each outcome	Y	Y	Y	Y	P	Y	Y

Ancillary analyses	P	N	I	N	N	N	N

Adverse events	P	P	Y	N	P	N	N

Interpretation	P	P	P	P	N	N	P

Generalisability	P	P	Y	P	N	N	N

Results placed into context	P	P	P	P	P	P	P

*Judged to be of sufficient quality for inclusion in explanatory meta-analyses?*	N	N	N	N	N	Y	Y for Oxford hip score only

**Key**: Y = Yes, included in paper/information to meet CONSORT level standards. N = not provided in paper/information. I = considered inappropriate/impossible.

P = Partially evident in paper/information. U = not fully provided/explained in paper/information and therefore unclear/ambiguous. *This trial was written up in summary form rather than as a journal paper.

Any initial disagreements regarding study quality were discussed until consensus was reached. Major disagreement was rare, usually disagreement was the more minor "yes" to "partial/unclear" or "no" to "partial/unclear" and 100% agreement was obtained. A third reviewer (CS) was available in the event of consensus not being reached but in the event this was not required. Where key study details were absent or unclear the authors were contacted for further information.

The quality of the studies evaluated in this review was mixed and generally poor (Table 2) with much relevant information initially missing from papers. Consideration was paid to the likelihood of serious potential bias being created throughout the assessment of quality decision making processes and this was taken into account when assessing individual trials. Only two papers were judged to be of sufficient quality to have been fully able to include in explanatory meta-analyses (with appropriate sensitivity analyses for blinding) if differing outcomes had not prevented these from occurring (Table 2). Problems existed within essential areas of trial quality in some trials. While one trial accurately identified itself as a non randomised trial [17] three others erroneously stated they were randomised trials. Two of these studies alternately assigned participants [18,19] which, since alternation is a non random "deterministic" approach [11], cannot be considered true randomisation. One further study assigned participants upon the basis of where they lived [20], another non random approach. Information provided regarding allocation concealment was inadequate for the majority of trials. Many studies either provided inadequate information regarding whether outcomes were measured by an assessor blinded to treatment allocation [17,21] or stated that such blind outcome measurement could not take place [22,23]. Whilst more recent trials were more likely to provide a justification of sample size, sample sizes were generally small (see Additional file 1) with most trials including fewer than forty subjects (range n = 20–58).

### Quantitative Data Synthesis

Despite the mixed and generally poor quality of the trials and their diverse outcomes we decided that it would be helpful to present a formal summary of the results where it was possible to perform such summaries. Sufficient data made this possible for both walking speed and hip abductor muscle strength but not function, range of joint motion or quality of life. For walking speed we first synthesised the results within in each study using the methods described by Gleser and Olkin (1994) [24] and we then combined these weighted mean differences to form a conventional fixed effects meta-analytic summary. Because we have no information about the correlation between measures within studies we carried out the analysis assuming a correlation of 0.8 and as a sensitivity analysis also used values of 0.2 and 0.5. For hip abductor muscle strength we carried out a conventional fixed effects analysis using standardised effect sizes. Since it is our object to present a summary rather than to encourage generalisability beyond these studies we neither used random effects nor present a formal assessment of heterogeneity. The assessment of publication bias was felt to be inappropriate due to the small number of trials available for inclusion in the review.

## Results

**Figure 1:**
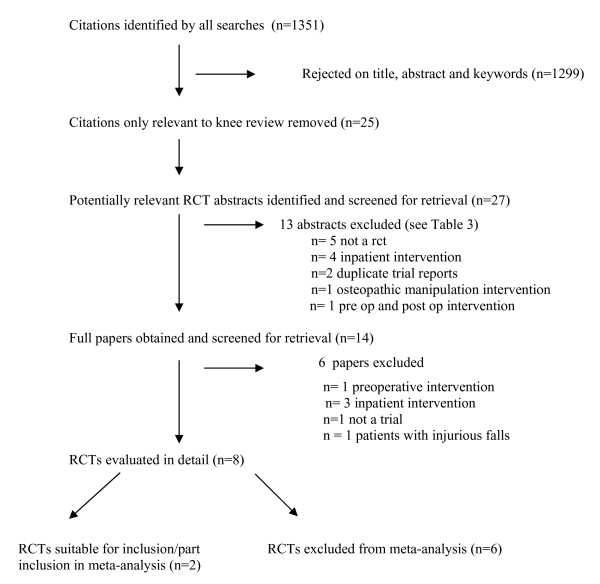
**Trial Flow diagram to summarise the stages of the systematic review**.

27 potentially relevant studies were identified and screened for retrieval. Of these, a total of 8 studies [16-23] were included in the systematic review. A summary is provided in the flow diagram in Figure 1. Table 3 contains a list of excluded studies [25-43].

**Table 3:** Studies Excluded from the Review.

**Reason for Exclusion**	**Study**
Not a clinical trial	Cullen *et al*., 1973 [25]
	Drabsch *et al*., 1998 [26]
	Freburger 2000 [27]
	Lapshin *et al*., 2002 [28]
	Richardson 1975 [29]
	Shih *et al*., 1994 [30]

In patient intervention	Chen *et al*., 2004 [31]
	Grange *et al*., 2004 [32]
	Hesse *et al*., 2003 [33]
	Hughes *et al*., 1993 [34]
	Lang 1998 [35]
	Maire *et al*., 2004 [36]
	Munin *et al*., 1998 [37]

Osteopathic manipulation intervention	Licciardone *et al*., 2004 [38]

Pre operative intervention	Gursen & Ahrens 2003 [39]

Pre op and post op intervention	Gilbey *et al*., 2003 [40]

Apparent multiple trial reports	Gilbey *et al*., 2003b [41] Werner *et al*., 2004 [42]

Inclusion criteria of injurious falls	Hauer *et al*., 2002 [43]

### Study Characteristics

The characteristics of the studies included in the trial are summarized in a table in Additional file 1. This table provides information regarding the participants, the interventions, the main outcomes and the conclusions reached by the authors.

### Summary of the Interventions and Comparisons

Details of the intervention and comparison groups were available from the papers and authors and these are summarised in a table in Additional file 2. The interventions provided to participants in the trials included in this review showed variation. One trial intervention consisted of a home exercise programme [17], one intervention was a home exercise programme with follow up visits to check and progress exercises [23], another also included a home exercise programme but with additional visits and telephone calls where necessary [18]. The exercises incorporated into trials also varied from addressing range of motion and strengthening [17,18] to targeting strength, postural stability and functional exercises [23].

The majority of trial interventions included some form of outpatient physiotherapy. This ranged from aerobic dance routines [20] to individualised physiotherapy treatment [16] to group training [19] to supervised strengthening sessions [22] and supervised exercising sessions plus home exercises [21]. The majority of trials allocated participants to intervention or control groups [16-18,20,21]. Three trials, occurring soon after surgery, compared the intervention group against an intervention based on usual care. These were described as traditional isometric and active range of movement exercises [23], a home exercise programme [19] and standard rehabilitation [22].

The timing of the intervention also varied. Some interventions started soon after surgery [22], and five weeks [16] and eight weeks [19] after discharge from hospital, while others took place up to several years post operatively [17,18].

The duration of interventions provided to trial participants ranged from 5–8 weeks [16,17,21,23] to programmes lasting around three to four months [18-20,22]. The frequency of physiotherapy ranged from daily [16,17,22] to 1–4 times per week [16,19-21,23]. Participants were usually followed up immediately post intervention with no long term follow up, except for Kaae et al, 1989 [16] who additionally included a 6 month follow up (additional file 1).

### Self Report measures of Function (5 trials, 190 participants)

Six studies reported results for functional activity measures [16-19,21,23]. The measures used included the following: The Oxford Hip Score [23], The McMaster Toronto Arthritis Patient Preference Disability Questionnaire (MACTAR) [19], the functional component of the Harris Hip Score [18], Unspecified activities of daily living/patterns of activity self report measures [16,21] and the Japanese Orthopaedic Score [17]. It can be seen that every study measured function differently and we felt that there was too much variety to usefully combine the results in a meta-analytic summary.

Within the individual trials, three demonstrated no observed significant differences between groups [16,19,21] while one trial used the score to describe the group characteristics at baseline [17]. Two recent trials [18,23] showed significant within group differences for the treatment arm only, indicating a treatment benefit within the treatment groups. Trudelle-Jackson and Smith, 2004[23] report a pre-intervention median Oxford Hip Score of 21 (range 15–33) and post-intervention median of 16 (range 12–38) for the treatment arm; Wilcoxon signed-rank test results revealed a significant difference *z *= -2.55, p = 0.01. Jan et al, 2004 [18] report functional component Harris Hip Score pre and post intervention means for their high compliance group of 11.7 (SD ± 0.8) and 13.1 (SD ± 0.6) respectively; Wilcoxon signed-rank test results reported a significant difference at p < 0.05 level. A personal communication from the authors reports the significant results (p < 0.05) for the exercise group as a whole; namely a pre intervention mean of 11.8 (± 0.8) and post mean of 12.9 (± 0.6).

### Walking (6 trials, 212 participants)

Some form of walking outcome measurement was used in six trials [16-21] although the means by which walking was measured varied. Mean "comfortable" walking speed over an unspecified time/distance measured in m/sec was measured in one trial [20]. Walking speeds in m/min were provided in two trials [17,18]. Jan et al, 2004 [18] measured fast and free walking speeds on hard and grass surfaces plus free walking speed measured on a spongy surface, whilst Sashika et al, 1996 [17] provided no further details of the test. Maximum walking speeds were provided in two trials [19,21]. Few procedural details were provided by Johnsson et al, 1998 [21] while Nyberg and Kreuter, 2002 [19] measured self selected maximum walking speed in seconds over a 30 m walkway. A twelve minute stamina walking test, without walking aids, on a treadmill was used by Kaae et al, 1989 [16]. In addition, cadence [17] and subjective gait analyses were included in one trial each [16].

The results from these trials are mixed. No significant differences were observed between groups in 2 trials [19,21]. Observed differences between groups were noted in walking stamina by Kaae et al, 1989 [16] and found to be significant in another trial (p < 0.05) [20]. In this trial, by Patterson et al, 1995, walking speed (m/sec) changed from a baseline mean of 1.28 (95% CI 1.18 to 1.38) to 1.41 (95% CI 1.31 to 1.51) post intervention for the intervention group and from 1.25 (95% CI 1.14 to 1.36) to 1.20 (95% CI 1.04 to 1.36) for the control group.

In addition, significant differences within interventions groups within a trial were observed within 2 trials [17,18]. Sashika et al, 1996 [17] report significant (p < 0.05) mean changes from 60.1 m/min to 63.6 m/min for group 1 (Table 1), and from 64.4 m/min to 69 m/min for group 2. Control group changes (non significant) were slower throughout from 57.4 m/min to 58.7 m/min. Jan et al, 2004 [18] report significant results for all forms of walking measured within the high compliance subgroup. However, within the exercise group as a whole, significant pre and post intervention differences (p < 0.05) were present for fast walking on level ground (pre intervention mean 86.8 m/min, SD ± 8.4 and post intervention mean 94.6 m/min, SD ± 16.8) and fast walking on grass (pre intervention mean 77.7 m/min, SD ± 8.1 and post intervention mean 84.4 m/min, SD ± 10.2). Free walking and walking on spongy surfaces results were not significant (personal communication).

**Figure 2:**
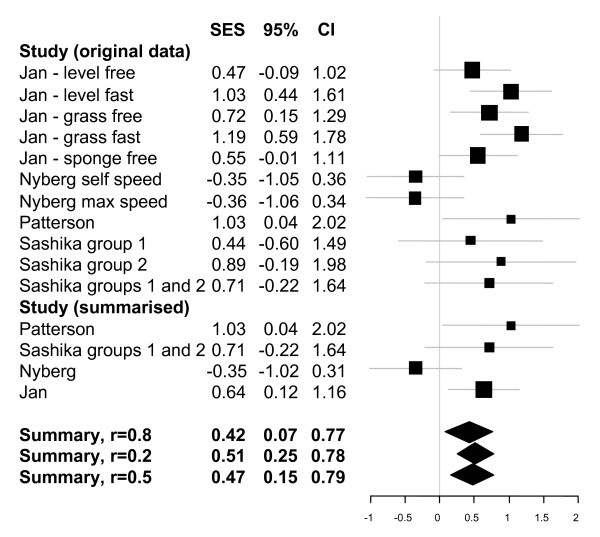
**Meta-analytic summary for walking speed**. Figure provides standardised effect sizes plus 95% confidence intervals.

Figure 2 presents the results of the quantitative analysis of walking speeds. The top part presents the results for each study for each measure considered, and for Sashika et al, 1996 [17] it shows the results of splitting the treatment group (as reported in the publication) as well as that of pooling it. The next part of the figure shows the summarised effect within each study (where appropriate). It is these four effects which are the subject of the meta-analysis. We have assumed that the correlation between each measure within each study was 0.8. The final part of the figure presents our summary values from the meta-analysis. The top one is for a correlation of 0.8 and the other two represent our sensitivity analysis assuming correlations of 0.2 or 0.5. As can be seen the substantive conclusion is similar but the smaller the within study correlation the more striking the overall summary appears and we have therefore chosen the more conservative value for our presentation.

### Range of joint motion (4 trials, 134 participants)

Range of motion was used as an outcome measure in four trials [16,17,19,21]. Johnsson et al, 1998 [21] measured passive hip flexion, extension defect, abduction and adduction. Nyberg and Kreuter, 2002 [19] measured passive hip flexion, extension, abduction and internal rotation using a goniometer for all measurements. Kaae et al, 1989 [16] provide few details regarding measurement. Sashika et al, 1996 [17] evaluated hip range of motion finding that hip flexion did not improve significantly within any groups. No significant differences between groups for hip joint range of motion were reported in the remaining three trials [16,19,21] and the lack of reported data prevented a useful meta-analytic summary.

### Muscle strength (6 trials, 207 participants)

Muscle strength was used as an outcome measure in six trials [16-18,21-23] and, once again, methodologies varied. Isometric quadriceps muscle force measurements, measured using dynamometry, were obtained by Suetta et al, 2004 [22], who used a maximal voluntary contraction approach, and Sashika et al, 1996 [17], who measured hip abductor maximal isometric torque. Johnsson et al, 1998 [21] also measured isometric muscle strength, of hip flexors, extensors, abductors and adductors and knee extensors and flexors, but used a different approach incorporating a strain gauge. Jan et al, 2004 [18] again used dynamometry, this time to measure isokinetic hip abductor, flexor and extensor muscle strength. Trudelle-Jackson and Smith, 2004 [23], used a BEP-IIIa force transducer and measured hip flexor, hip extensor, hip abductor and knee extensor muscle strength via a "make test". Finally both Kaae et al, 1989 [16] and Sashika et al, 1996 [17] used manual muscle testing.

In summary, no differences between groups were observed in 2 studies [16,21]. Statistically significant differences (p < 0.05) between and within groups were observed in one study [22]. Baseline quadriceps isometric strength mean values (operated leg) were 122.9 Nm (SE ± 17.2) for the intervention group and 119.2 (SE ± 15.9) for the standard rehabilitation group. At 12 weeks these values had changed to 119.2 Nm (SE ± 17.8) for the intervention group and 117.4 (SE ± 13.8) for the standard group. Between group significant differences (p < 0.05) were also present for vastus lateralis mean average voltages, using EMG, again in favour of the intervention group. Within group significant differences were also observed within the intervention group for contractile rapid force development (26–45% increase p < 0.05) and contractile impulse (27–32% p < 0.05).

In addition, statistically significant differences, within intervention groups, were observed in 2 studies [18,23]. Trudelle-Jackson and Smith, 2004 [23] report muscle strength (Nm) percentage change from baseline to post intervention. Within the intervention group, hip flexors = 24.4%, hip extensors = 47.8, hip abductors = 41.2% and knee extensors = 23.4%; all statistically significant changes (p < 0.05) which demonstrated improved muscle strength. Whereas no significant differences occurred within the control group; hip flexors = 7.2% change, hip extensors = 3.6%, hip abductors = 3.3% and knee extensors = 1%. Jan *et al *(2004) [18] demonstrated hip abductor, flexor and extensor muscle strength (operated side) to be significantly different (p <0.05) between pre and post intervention time points within the exercise group but not within the control group (personal communication). Hip abductor mean values were 53.5 Nm (SD ± 18.4) pre intervention and 59.9 (SD ± 18.2) post intervention for the intervention group and 55.7 (SD ± 17.7) and 52 (SD ± 21) for the control group. Hip flexor strength was 49.2 Nm (SD ± 19.2) pre intervention and 54.5 (SD ± 17) post intervention within the intervention group and 54.2 (SD ± 22.5) and 50.8 (SD ± 21.2) within the control group. Hip extensor mean values were 71.4 Nm (SD ± 23.4) pre intervention and 76.1 (SD ± 24.9) post intervention for the intervention group and 74.8 (SD ± 31.1) and 72.5 (SD ± 24.2) for the control group. When the results were further broken down to reflect high and low compliance subgroups [18], both operated and non operated sides demonstrated significantly different pre and post intervention values within the intervention group.

**Figure 3:**
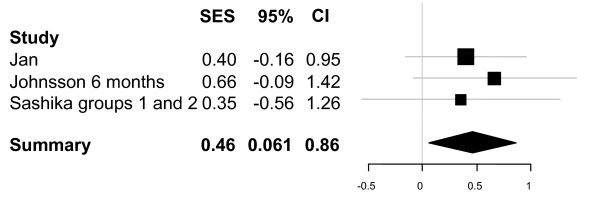
**Meta-analytic summary for hip abductor muscle strength**. Figure provides standardised effect sizes plus 95% confidence intervals.

Figure 3 presents the results of the quantitative analysis of hip abductor muscle strength for studies including sufficient data. The summary suggests that physiotherapy exercise shows promise in increasing hip abductor strength for this patient group. There were insufficient studies including hip extensor and hip flexor strength to make meta-analytic summaries useful for these muscle groups.

### Quality of Life (1 trial, 55 participants)

A 0–100 mm visual analogue scale to measure quality of life was used in one trial [19]. No significant differences between the groups were demonstrated.

## Discussion

### Summary of principal findings

This systematic review finds that it is not yet possible to establish the extent to which post discharge physiotherapy exercise is effective, in terms of improving function, quality of life, mobility, range of hip joint motion and muscle strength, for osteoarthritic patients following elective primary unilateral total hip arthroplasty. The diversity and lack of available trials plus the unsatisfactory quality of existing trials prevent a definitive answer at this time. Trials provided mixed results and it would also be wrong to conclude at this time that post discharge physiotherapy is ineffective, especially since the meta-analytic summaries of the data indicate promising potential benefit.

### Strengths and weaknesses of review procedures

The reviewers believe the search strategy to be comprehensive and to have been successful in locating relevant trials for inclusion in the review. Physiotherapy literature remains a difficult area to search, with numerous bibliographic data bases and un-indexed journals [44] and while every attempt was made to identify studies it is possible other studies exist. However, this review remains the most comprehensive to date. The two non English language trials identified in the literature search were professionally translated to allow full inclusion and to prevent the introduction of language bias [45,46].

The quality of trials was mixed and generally poor. The trials included in the review spanned nearly thirty years and it was noticeable that the more recent trials reported necessary information more clearly and comprehensively than trials published many years previously. This may not be a reflection on the quality of earlier trials as such, rather it may be that reporting habits and editorial requirements have altered and improved over recent years, particularly since the introduction of the CONSORT statement. Similarly, more recent trials were more likely to justify their sample sizes and comment upon the power of their studies than earlier ones. The literature search for this review included trials up to April 2007 and is considered up to date.

There were no apparent problems with the data extraction processes used in this review. Although many quality checklists and scales exist, there is no accepted "gold standard" score; component approaches are often preferred since the wide variety of scores and weighting systems available mean that the same trial may score as both high quality and low quality depending on which score is used [15]. Additionally, many scoring systems downgrade the quality rating of a trial if it is not double blinded. For many physiotherapy trials, such as those in this review, it is inevitable that patients and therapists know whether they are receiving the physiotherapy intervention or the control and this is not an indication of low/high trial quality. For these reasons, as previously [14], we used a component approach although we accept this is a controversial area of debate.

The independent reviewers showed good percentage agreement with each other and moderate agreements when using Cohen's kappa [47] and the Intraclass Correlation Coefficient [48]. This level of initial agreement was considered acceptable since interpretation of some checklist items, such as generalisability and overall evidence, can be subjective. Following discussion both reviewers were in full agreement for all items for all papers.

Where possible, a comprehensive description of trials interventions has been presented for the majority of trials (additional file 2). It was not possible to obtain such details for all trials, particularly early trials where it was not possible to track down author contact details. As is often the case with physiotherapy trials [49], the studies are small with 282 participants in eight studies. The number of available studies, and their size and quality, does limit this review. It is perhaps surprising that so few published trials exist for such a common and longstanding area of physiotherapy practice. This would seem to be partially attributable to the general lack of rehabilitation research undertaken on orthopaedic surgery patients post discharge, rather than hip replacement patients per se, since a recent search for knee replacement trials by the authors revealed a similar lack of trials [14].

It was not possible to include pain as a main outcome in this review since the studies identified in this review did not tend to measure pain as a specific outcome. This does not mean that pain is considered unimportant. Some available functional measures include a pain subscale while others, such as the Oxford hip score, include pain as a component within the score. The influence of pain on the performance of objective measures also needs to be considered. However, the means by which pain is measured may, as in this review, make pain difficult to explore systematically across studies.

We have tried to summarise the data fully, using meta-analytic summaries where the data enabled us to do so appropriately [24] (Figures 2, 3). These figures are intended to helpfully summarise the data only; we emphasise that the mixed quality and diversity of trials in this review prevented explanatory meta-analyses from being undertaken, since the results would risk being misleading or erroneous, and we do not intend these figures to be interpreted in this way.

### Clinical implications

This review cannot remove the current uncertainty regarding post discharge physiotherapy for this patient group, although it is useful in summarising the current available research, in highlighting the lack of existing evidence and demonstrating the need for such evidence to be obtained. Commissioning organisations, health care practitioners and patients still lack conclusive evidence regarding effectiveness when deciding whether to provide/attend post discharge physiotherapy following elective primary hip replacement for osteoarthritis. A systematic review including meta-analyses for a similar question following knee replacement [14] provided support for the use of physiotherapy functional exercise interventions following discharge to obtain short term benefit to patients following elective primary knee arthroplasty. Whilst differences between suitable activities and rehabilitation following joint replacement are recognised for knee and hip patients, many similarities remain [9] and these results contribute to the argument for further trials for hip replacement patients being necessary.

### Future directions

The three trials incorporating home exercise programmes [17,23,23] demonstrated pre-post intervention differences within the intervention groups but not in the controls. These trials were for participants who had had their hip replacements some time previously rather than for people recently discharged from hospital. Trials comparing differences between groups would be valuable. As would research to explore the optimum time-point at which to offer any additional post discharge physiotherapy exercise intervention to patients. The trials which included out-patient individual or group training following discharge tended to report between group differences with generally negative results [16,19,21] however the temptation to count up the number of these negative trials, rather than await future high quality trials, should be avoided. Across the entire body of hip replacement knowledge, research indicates that hip replacement patients experience persistent functional and physical limitations at least one year post-operatively [8] when many follow up studies cease obtaining outcome data. Whether physiotherapy exercise post discharge can reduce such limitations therefore remains an important question to adequately address. Recent research indicates that traditional physiotherapy following lower limb joint replacement, consisting of range of joint motion and isometric muscle strengthening exercises plus transfer and gait/walking aid practice, may also be less effective than programmes incorporating a more functional, weight bearing approach to rehabilitation [7,23,50]. We believe it is both timely and necessary for well conducted clinical trials investigating the effectiveness of physiotherapy exercise interventions following discharge after elective primary total hip replacement surgery to take place for this common area of clinical practice.

## Conclusion

In conclusion, insufficient evidence currently exists to establish the effectiveness of physiotherapy exercise following primary hip replacement for osteoarthritis. Further well designed trials are required to determine the value of post discharge exercise following this common surgical procedure.

## Competing interests

The authors declare that they have no competing interests.

## Authors' contributions

CJML lead and designed the review, carried out searches and eligibility checks, reviewed and extracted data, performed qualitative analysis and drafted the manuscript. KB assisted in designing the review, served as a blind reviewer, extracted data, performed qualitative analysis and commented upon the draft manuscript. MED designed and carried out the quantitative data analysis and commented upon the draft manuscript. CMS assisted in designing the review, eligibility checking, and commented upon the draft manuscript.

All authors read and approved the final manuscript.

## Pre-publication history

The pre-publication history for this paper can be accessed here:



## Supplementary Material

Additional file 1 **Study Characteristics of the Trials Evaluated in the Systematic Review (n = 8)**. Table summarising the study characteristics of the trials included in the systematic review. Click here for file

Additional file 2 **Summary of trial interventions and comparisons included in the hip replacement trials (n = 8)**. Table summarising the interventions provided to participants within each of the trials included in the systematic review Click here for file
