# Supplementary material for: Effectiveness of physiotherapy exercise following hip arthroplasty for osteoarthritis: a systematic review of clinical trials
Source: BMC Musculoskelet Disord. 2009 Aug 4;10:98. doi: 10.1186/1471-2474-10-98 (PMC2734755; doi:10.1186/1471-2474-10-98)
Supplement: Additional file 1 — Study Characteristics of the Trials Evaluated in the Systematic Review (n = 8). Table summarising the study characteristics of the trials included in the systematic review. [file 1471-2474-10-98-S1.doc]

| **Paper** | **Participants (sample size)** | **Intervention ( and time of intervention)** | **Main Outcome Measures (and time/s of follow up)** | **Results** |
| --- | --- | --- | --- | --- |
| Jan *et al.,* 2004 | Unilateral primary THR patients (58) | Comparison of a 12 week home exercise programme (with phone calls and extra visits if necessary) versus control group (at least 1.5 years post operatively) | Muscle strength. Walking speed. Functional ability. Compliance.  (Follow up: post intervention at 12 weeks) | Exercise group showed significant within group pre-post intervention difference (P<0.5), control group did not. Further analysis showed “high compliance exercisers” showed significantly greater (P<.05) improvement in outcome measures than “low compliance exercisers” and control. |
| Johnsson *et al.,* 1998 | Unilateral primary THR (30) patients. | Intervention group received organised OPD PT exercises for 1-2 months versus control group (2 months post operatively) | Passive hip mobility. Maximum  *isometric* muscle strength. Limb length discrepancy. Walking speed. Trendelenberg. Limp. Walking supports. Stair climbing. Sitting & rising from chair. ADL. (Follow up: 6-8 wks post operatively and 6 months) | No significant differences between the groups for any of the outcome measurements. |
| Kaae *et al.,* 1989 | Unilateral THR patients (26) | 5 week intervention of OPD PT (mobility, gait and posture training plus any individual treatment needs) three times a week (5 weeks post operatively) | Joint mobility. Hip Strength. Postural stability. Stamina in walking. Gait. Borg Scale. ADL questionnaire.  (Follow up: 2 ½ and 6 months post operatively) | Differences between gps for stamina, gait, security without sticks, ADL and Borg values, favouring intervention. No differences for joint mobility, strength & postural stability. |
| Nyberg and Kreuter, 2002 | Primary THR patients (55) | All taught home training programme. Treatment groups had additional group training twice a week for 15 weeks. Training included movement training, muscle training and walking exercises (8 weeks post operatively) | MACTAR instrument. Hip joint range of motion. Walking speed. Pain on activity. Quality of life. Opinion on training.  (Follow up: post intervention at 6 months post operatively) | No significant differences were observed between the two groups in any of the measured variables. |
| Patterson *et al.,* 1995 | Unilateral or bilateral primary female THR patients (20) | Comparison of intervention (exercise programme, including aerobic-dance routines, for three months)  versus control (6 months post operatively) | Exercise intensity. Treadmill test (Peak VO_2_ in ml/kg/min, Peak VO_2_ in Peak heart rate, Peak respiratory exchange ratio, Exercise duration, VO_2_ at anaerobic threshold, Peak lactate, VO_2_ at lactate threshold. Blood pressure. Heart rate. ST segment displacement, end tidal CO_2_ , VCO_2_, VE anaerobic threshold. Borg scale. Walking speed. Body mass. Skin folds. Body fat. (Follow up: post intervention at 3 months) | Peak VO_2_ increased in the intervention gp compared to baseline (p<.05) but did not differ significantly from control. Intervention gp significantly increased their walking speed by 10.1% compared with baseline and control (p<.05), and also showed increased VO_2_ at lactate threshold (compared to baseline and control). |
| Suetta *et al.,* 2004 | Unilateral primary THR patients (36). | Comparison of 3 groups.  1. standard rehabilitation (SR) 1 h/d for for 12 wks  2. standard rehabilitation plus unilateral strength training 3/wk for 12 wks(ST)  3. SR plus neuromuscular electrical stimulation (NMES) - this arm excluded from systematic review) (post operatively 1h/d for 12 wks) | Quadriceps muscle cross sectional area.  Maximal voluntary isometric quadriceps strength. Rapid muscle force development.  EMG to evaluate changes in muscle activation.  (Follow up: 5 and 12 weeks post operatively) | In the ST gp significant differences were observed in maximal isometric muscle strength (24% p<.01); contractile rapid force development (26-45% p<.05) and contractile impulse (27-32%, p <.05). Mean EMG signal amplitude for vastus lateralis was larger in ST gp than SR gp at 5 and 12 weeks after surgery (p<.05). No within gp significant differences observed for the other gps. |
| Sashika *et al.,* 1996 | THR patients -including revisions and re-revisions (23) | 6 wk home programme. 3 gps. Gp 1 = Range of motion & low resistance isometric muscle strengthening . Gp 2 = all Gp 1 ex plus eccentric hip abductor muscle strengthening. Gp 3 = control ( Mean 26.4 months post operatively) | Range of hip motion. Muscle strength. Hip abductors Maximal isometric torque. Gait speed. Cadence. Japanese orthopaedic score. Compliance.  (Follow up: post intervention at 6 weeks) | Significant improvements within groups for maximum isometric torque (Gp 1 = p<0.01 THR side; Gp 3 p<.05 THR side and both sides for gp 2 p<.01). Gait speed and cadence also improved within gps 1 and 2. |
| Trudelle Jackson and Smith, 2004. | THR patients (34) | 2 groups for 8 week programme 4-12 months post surgery. Intervention = strength and postural stability exercises versus Traditional isometric and active range of movement ex ( 4-12 months post operatively) | Oxford hip scores. Muscle strength. Postural stability. Fear of falling. Compliance.  (Follow up: post intervention at 8 weeks) | Statistically significant results were observed for all outcome measures in the intervention gp, except fear of falling. No significant differences were observed within the traditional gp. |

Key – THR =denotes total hip replacement; h/d= hour/day; OPD PT= out patient department Physiotherapy; Gp= group; ADL=activities of daily living.
