# Supplementary material for: Effectiveness of physiotherapy exercise following hip arthroplasty for osteoarthritis: a systematic review of clinical trials
Source: BMC Musculoskelet Disord. 2009 Aug 4;10:98. doi: 10.1186/1471-2474-10-98 (PMC2734755; doi:10.1186/1471-2474-10-98)
Supplement: Additional file 2 — Summary of trial interventions and comparisons included in the hip replacement trials (n = 8). Table summarising the interventions provided to participants within each of the trials included in the systematic review [file 1471-2474-10-98-S2.doc]

|  | **Early programme provided to all participants** | **Intervention** | Comparison |
| --- | --- | --- | --- |
| Jan *et al.,*  2004 |  | *Range of motion*: Hip & Knee flexion and extension. *Strength* (with 1 kg ankle weight for women, 2 kg for men) Supine: Hip flexion a) with knee flexed b) with knee extended. Prone: Hip extension. Side lying: Hip abduction. Exercises in standing: Alternate single leg stance (5 s hold) 10 reps X 2, daily. 30 mins walk | Nil else added |
| Johnsson *et al.,* 1998 | 20 mins physiotherapy session days 7- 12 post operation. Supine: Straight leg raise, hip extension against resistance, hip abduction. Sitting: Knee flexion, extension Gait: stick/s. Advice: regarding sitting/bending; aids, raised toilet seat, seat cushion. Drive, cycle, swim from 6-8 wks post operation. | *Outpatient physiotherapy*. Supine: activated abdominals, hamstrings and quads by 1) lift pelvis with lower limb resting on cushion. 2) lift pelvis holding a ball between flexed knees keeping feet on floor. Repeat 1&2 weightbearing on 1 leg alternately. Four point kneeling: alternate leg extension. Standing: weight shift side-to-side in different directions to activate gluts, hams, hip abductors, tenscia fascia lata, iliopsoas, quadriceps and gastrocnemius, standing on toes: whilst flexing hips and knees. Single leg stand: swing other leg slow backwards and forwards. St: step up/down alternate leg .Sitting: sit to stand (weight bearing mostly 1 leg at a time to activate gluteal, hamstrings, tenscia fascia lata, iliopsoas and quadriceps). Walking programme: 2Xwk for 1/12. Then 1X wk for 1/12 OR 1X wk for 2/12. Duration 45 mins. Do until 6/12 follow up |  |
| Kaae *et al.,* 1989 | Inpatient exercise programme plus home exercise programme after discharge. | Out patient physiotherapy after discharge: mobility training, gait & posture correction and to address individual needs as appropriate. Treatment from around 5/52 post operation, around 45 mins 3 X a week for 5 weeks |  |
| Nyberg & Kreuter, 2002 | Inpatient: gait training with 2 crutches, weight bearing as pain allows, range of movement exercises. Visit to physiotherapist 8 wks post op for exercises. Standing: unilateral hip extension, abduction plus ankle dorsiflexion. Repeat alternate leg. Bilateral heel raises. Standing at wall: gentle squats. Sitting: knee extension, knee lifts. | *Movement training* in standing and lying and *muscle training* against the limb’s own weight and walking in standing and lying. Walking exercises.  Group training 2 X wk for 15 weeks. Each session approximately 45 mins | Continued with home training until follow up at 6 months post op |
| Patterson *et al.,* 1996 |  | *Warm up* 5-10 mins (walking, flexibility). *Aerobic* dance to music (15-20 mins)  *Cool down*: stretches & balance exercises (10 mins). Short relaxation periods built in which decreased as fitness improved. 2X per week for 12/52s at least 6/12 post operation | No programme- normal activities only |
| Sashika *et al.,* 1996 |  | Group A. *Range of movement*, supine: hip flexion. All fours: hip flexion. Sitting: hip flexion. *Strength ex*e*rcises* (using ankle weight 20-30% of maximum isometric muscle torque), Supine: for hip flexors. Side Lying: for hip abductors. Prone: for hip extensors. Sitting: for knee extensors. Group B. as for Gp A plus 1 *strength exercise* in standing (no ankle weight): 1 leg stand: elevating pelvis for hip abductors. 2x day for 6/52 | Control group had No programme |
| Suetta *et al.,* 2004 | Standard rehabilitation booklet. Bed exercises: ankle dorsiflexion, plantarflexion, isometric gluts, pelvic & thigh muscles. Sitting: knee extension. Standing: hip abduction, knee flexion, step training & calf stretches. 2 X daily. | *Progressive quadriceps training.* *In patient,* sitting: daily unilateral knee extension (3X10 reps) with sandbags strapped to ankle. *From day 7*, 10 mins static cycling warm up, supine: leg press & knee extensor gym machine training 3 X wk. *Training intensity* increased from 20 RM max (~50% if 1RM) in wk 1; 15 RM (~65% of 1RM) in wks 2 to 4; 12RM (~70% of 1RM) in weeks 5-6; 8RM (~80% of 1RM) for last 6 wks. Wks 1-6: 3 -5 sets of 10 reps. Weeks 6 to 12: 5-8 sets of 10 reps. Exercise done quickly in concentric phase and slowly during eccentric phase. Training load adjusted weekly | Control session 1 X wk: Physiotherapist checked home exercises |
| Trudelle-Jackson & Smith, 2004 |  | Sitting: sit to stand. Standing: Unilateral heel raises, partial knee flexion, single leg stand, knee raises with alternate arm raises, side & back leg raises, unilateral pelvic raising & lowering. Repetition rate (RR) =15, 3-4 X wk for 8 wks. If able RR increased to 20 at 1^st^ follow up (2wks) and 2X20 at 2^nd^ follow up (8 wks). Control and quality of movement emphasised throughout. | Gluts, Hamstrings and quadriceps sets, ankle pumps, heel slides. Sup: hip abduction, internal rotation and external rotation. RR as for intervention group. |
